# Prediction of [^177^Lu]Lu-DOTA-TATE therapy response using the absorbed dose estimated from [^177^Lu]Lu-DOTA-TATE SPECT/CT in patients with metastatic neuroendocrine tumour

**DOI:** 10.1186/s40658-024-00620-8

**Published:** 2024-02-05

**Authors:** Sejin Ha, Yong-il Kim, Jungsu S. Oh, Changhoon Yoo, Baek-Yeol Ryoo, Jin-Sook Ryu

**Affiliations:** 1 Department of Nuclear Medicine, Asan Medical Center, University of Ulsan College of Medicine, Seoul, Republic of Korea; 2 Theranostics Center, Asan Cancer Institute, Asan Medical Center, Seoul, Republic of Korea; 3 Department of Oncology, Asan Medical Center, University of Ulsan College of Medicine, Seoul, Republic of Korea

**Keywords:** Neuroendocrine tumour, [^177^Lu]Lu-DOTA-TATE, SPECT/CT, Dosimetry, Absorbed dose

## Abstract

**Background:**

Peptide receptor radionuclide therapy (PRRT) with [^177^Lu]Lu-DOTA-TATE has shown efficacy in patients with metastatic neuroendocrine tumours (NETs). Personalised dosimetry is crucial to optimise treatment outcomes and minimise adverse events. In this study, we investigated the correlation between the tumour-absorbed dose (TAD) estimated from [^177^Lu]Lu-DOTA-TATE SPECT/CT and the therapeutic response.

**Method:**

A retrospective analysis was conducted on patients with advanced well-differentiated NETs grades 1–3 who underwent PRRT and exhibited greater uptake than liver on pre-therapeutic [^68^Ga]Ga-DOTA-TOC PET/CT. Target lesions were selected based on the RECIST 1.1 and PERCIST 1.0 criteria using [^177^Lu]Lu-DOTA-TATE SPECT/CT and pre-therapeutic contrast-enhanced CT scans. For anatomical image analysis, the sum of the longest diameter (SLD) of the target lesions was measured using the RECIST 1.1 criteria for patient-based analysis and the longest diameter (LD) of the target lesion using the RECIST-L criteria for lesion-based analysis. Standardised uptake values (SUVs) were measured on SPECT/CT images, and TADs were calculated based on the SUVs. Dosimetry was performed using a single SPECT/CT imaging time point at day 4–5 post-therapy. Statistical analyses were conducted to investigate correlations and determine the target lesion responses.

**Results:**

Twenty patients with primary tumour sites and hepatic metastases were included. Fifty-five target lesions, predominantly located in the pancreas and liver, were analysed. The cumulative TAD (lesion-based analysis: *r* = 0.299–0.301, *p* = 0.025–0.027), but not the cycle 1 SUV (lesion-based analysis: *r* = 0.198–0.206, *p* = 0.131–0.147) or cycle 1 TAD (lesion-based analysis: *r* = 0.209–0.217, *p* = 0.112–0.126), exhibited a significant correlation with the change in LD of the target lesion. Binary logistic regression analysis identified the significance of the cumulative TAD in predicting disease control according to the RECIST-L criteria (odds ratio = 1.031–1.051, *p* = 0.024–0.026).

**Conclusions:**

The cumulative TAD estimated from [^177^Lu]Lu-DOTA-TATE SPECT/CT revealed a significant correlation with change in LD, which was significantly higher for the cumulative TAD than for the cycle 1 SUV or TAD. A higher cumulative TAD was associated with disease control in the target lesion. However, considering the limitations inherent to a confined sample size, careful interpretation of these findings is required. Estimation of the cumulative TAD of [^177^Lu]Lu-DOTA-TATE therapy could guide the platform towards personalised therapy.

**Supplementary Information:**

The online version contains supplementary material available at 10.1186/s40658-024-00620-8.

## Background

^177^Lu-DOTA-0-Tyr3-Octreotate ([^177^Lu]Lu-DOTA-TATE, Lutathera®) therapy, a peptide receptor radionuclide therapy (PRRT) targeting the somatostatin receptor (SSTR), is known to be effective in patients with metastatic neuroendocrine tumours (NETs) [1–5]. Personalised dosimetry of [^177^Lu]Lu-DOTA-TATE therapy is potentially effective in maximising its therapeutic effects and minimising adverse events [6, 7]. [^68^Ga]Ga-DOTA-TOC uptake is commonly used to assess the feasibility of PRRT and select suitable candidates by targeting SSTR [8]. Certain studies have indicated a notable link between pre-therapeutic [^68^Ga]Ga-DOTA-TOC uptake and the absorbed dose in [^177^Lu]Lu-DOTA-TATE therapy, suggesting that increased [^68^Ga]Ga-DOTA-TATE uptake is associated with higher absorbed doses during [^177^Lu]Lu-DOTA-TATE therapy. However, this relationship may not be robust enough for individualised dose planning [9, 10]. One study demonstrated a relatively strong correlation between the tumour-absorbed dose during [^177^Lu]Lu-DOTA-TATE therapy and tumour reduction [11]. However, other studies have shown no significant correlation between the tumour-absorbed dose and tumour reduction [12, 13].

The absorbed dose of [^177^Lu]Lu-DOTA-TATE by the tumour was originally estimated by performing 4 to 5 repeated sessions of [^177^Lu]Lu-DOTA-TATE scintigraphy; however, this is too difficult to routinely perform in clinical practice. As an alternative, single-photon emission computed tomography/computed tomography (SPECT/CT) performed 4–5 days after PRRT can be used to accurately measure the absorbed dose of [^177^Lu]Lu-DOTA-TATE by the tumour [14–16].

We hypothesised that the tumour-absorbed dose estimated from single SPECT/CT performed 4–5 days after PRRT could predict tumour response. In this study, we identified a correlation between the tumour-absorbed dose estimated from [^177^Lu]Lu-DOTA-TATE SPECT/CT and the therapeutic response of the tumour according to the diameter changes on CT.

## Methods

### Patient selection

**Fig. 1:**
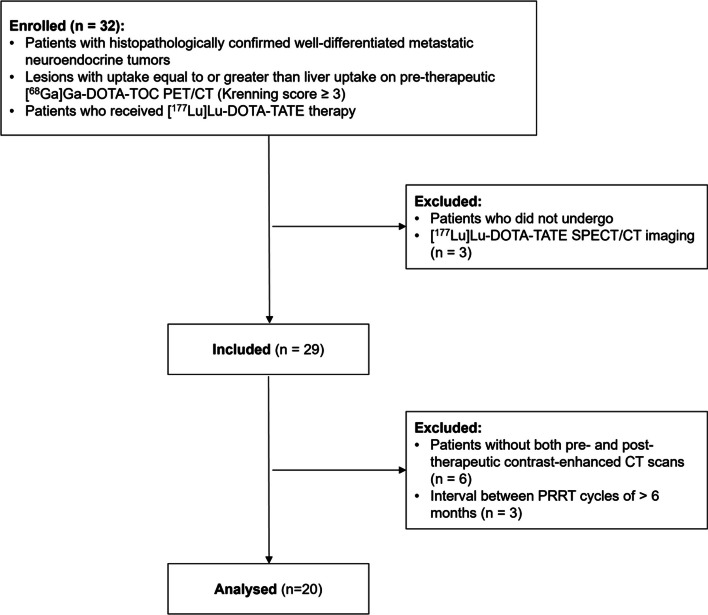
Flow diagram of patient inclusion and exclusion

Between December 2019 and December 2021, a total of 32 patients with metastatic NETs who underwent PRRT were retrospectively evaluated. Patients were considered suitable for PRRT if they had advanced, well-differentiated NET grades 1–3, with more uptake than the liver on pre-therapeutic ^68^Ga-DOTA-D-Phe1-Tyr3-Octreotide ([^68^Ga]Ga-DOTA-TOC) positron emission tomography (PET)/CT (Krenning score ≥ 3, on maximum intensity projection images) [17]. Patients who underwent [^177^Lu]Lu-DOTA-TATE SPECT/CT 4–5 days after PRRT scans were included. Six patients without either pre-therapeutic contranst-enhanced CT (CECT) or post-therapeutic CECT and three patients with intervals greater than 6 months between PRRTs were excluded from the study (Fig. 1). The other inclusion criteria were as follows: age > 18 years, Karnofsky performance status (KPS) ≥ 70, estimated glomerular filtration rate (eGFR) > 40 mL/min, creatinine ≤ 1.7 mg/dL, haemoglobin (Hb) > 8 g/dL, white blood cell (WBC) count > 2 000/μL, platelet count (PLT) > 70 000/μL, and total bilirubin < 3.0 mg/dL. This study was approved by our Institutional Review Board (IRB No. 2022-1581), and the requirement for informed consent was waived.

### PRRT and SPECT/CT

Every 2–3 months, 7.4 GBq of [^177^Lu]Lu-DOTA-TATE (Lutathera®, Norvatis, Switzerland) was intravenously injected up to four times [18]. Short- and long-acting somatostatin analogues were discontinued 24 h and 4 weeks before every treatment, respectively. On the day of therapy, patients were fasted for 4 h before and 2 h after PRRT. Two intravenous (IV) lines were inserted and vital signs and peripheral oxygen saturation (SpO_2_) checked. The antiemetic drug ondansetron (Zofran®; GlaxoSmithKline, United Kingdom) was medicated 1–2 h before [^177^Lu]Lu-DOTA-TATE infusion. Next, 1 000 mL of L-arginine 25 g/L-lysine 25 g (LysaKare®; Advanced Accelerator Applications, a Novartis company, France) was infused at a rate of 250 mL/h at least 30 min before initiation of [^177^Lu]Lu-DOTA-TATE infusion. [^177^Lu]Lu-DOTA-TATE was infused at a rate of 60 mL/h for approximately 30 min using a syringe pump. Patients were closely monitored during and for 4 h after PRRT to observe any acute side effects, including flushing, nausea, vomiting, diarrhoea, bronchospasm, hypertension, and carcinoid crisis. The patient’s condition, including vital signs and SpO_2_, was also recorded on a sheet every 30–60 min.

SPECT/CT imaging was performed 4–5 days after PRRT. Images were acquired using an integrated SPECT/CT scanner (Symbia Intevo; Siemens, Germany) equipped with medium-energy, low-penetration collimators from the neck to the proximal thigh area. The CT was acquired using the following parameters: 110 kVp, 40 ref mAs using adaptive dose modulation (CARE Dose 4D), 16 × 0.6 collimation, 1-s rotation time, 2-mm slice thickness, 2-mm increment, and 1 pitch. SPECT was acquired using the following parameters: 20% energy window centred at 208 keV, 256 × 256 matrices, 1.0 × zoom, 45 view, 22 s per view, and step-and-shoot mode. Image reconstruction was performed using an ordered subset conjugate gradient minimiser (OSCGM) algorithm (xSPECT; Siemens) with 24 iterations, 2 subsets, 5-mm Gaussian filter, and 256 × 256 matrices, enabling the quantification of SPECT/CT images. Our SPECT/CT camera utilized Siemens xSPECT software capable of producing SUV images, unlike traditional SPECT images that display counts, thereby enhancing the accuracy of our dosimetry study.

### Target lesion selection

Target lesions were selected according to the RECIST 1.1 and practical PERCIST 1.0 criteria [19–21]. Tumours with the hottest uptake on [^177^Lu]Lu-DOTA-TATE SPECT/CT with > 10 mm in the longest diameter on pre-therapeutic CECT scan were selected. Up to five tumours per patient and up to two tumours per organ were analysed. Bone metastases were excluded because the diameter change of bone metastases cannot be appropriately evaluated with CECT [22–25].

### Anatomical image analysis

The longest diameter of the target lesion was measured on pre- and post-therapeutic CECT and averaged by two experienced nuclear medicine physicians (S.J.H. and Y.I.K.) who were blinded to the clinical and SPECT/CT data. Pre-therapeutic CECT scans were performed within 3 months prior to cycle 1 PRRT, with a median of 29 days (range: 3–74 days). Post-therapeutic CECT scans were performed within 3 months after the final PRRT cycle, with a median of 30 days (range: 2–71 days). Changes in the diameters of target lesions were measured and evaluated by patient-based and lesion-based according to the RECIST 1.1 and RECIST-L criteria, respectively [20].

1. RECIST 1.1 (patient-based) criteria: disappearance of the target lesion was defined as ‘complete response’, decrease in the sum of the longest diameters (SLD) of the target lesions ≥ 30% was defined as ‘partial response’, increase in the SLD of the target lesions ≥ 20% was defined as ‘progression’, and in between was defined as ‘stable disease’.
2. RECIST-L (lesion-based) criteria: disappearance of the target lesion was defined as ‘complete response’, decrease in the longest diameter (LD) of the target lesion ≥ 30% was defined as ‘partial response’, increase in the longest diameter of the target lesion ≥ 20% was defined as ‘progression’, and in between was defined as ‘stable disease’.

Disease control was defined as ‘partial response’ or ‘stable disease’. Both patient-based and lesion-based analyses were performed.

### Standardised uptake value (SUV) measurement

The SUVs of target lesions were measured on SPECT/CT images 4–5 days after treatment with [^177^Lu]Lu-DOTA-TATE using Mirada DBX software (version 1.2.0.59; Mirada Medical, Ltd., Oxford, United Kingdom) [26–28]. SUV_max_ was defined as the voxel with the highest uptake on SPECT/CT within the volume-of-interest (VOI) of RECIST 1.1-selected lesions. The SUV_peak_ was defined as the maximum average SUV within a 1-cm^3^ sphere. The SUV_41_ was defined as the mean SUV of all voxels with an activity of 41–100% of the voxel with the highest uptake (SUV_max_) within the VOI. The SUV_41_ was calculated by adjusting the iso-contour, which was automatically delineated using 41% of SUV_max_ within the VOI [29]. Most target lesions were automatically delineated; nevertheless, a few VOIs of the target lesions required manual correction to exclude other closely located tumour lesions.

### Tumour-absorbed dose (TAD)

Based on a study by Hanscheid et al. [14], we used the SUV_max_, SUV_peak_, and SUV_41_ of target lesions measured on SPECT/CT taken 4–5 days after PRRT by converting them to Dose_max_, Dose_peak_, and Dose_41_, respectively. Unlike Hanscheid et al., who formulated the absorbed dose in terms of counts in the VOI, we formulated the absorbed dose in terms of SUV as follows:

The equation used by Hanscheid et al. can be rephrased as [14]:

$$D\left[Gy\right]=0.25 \left[Gy \frac{g}{MBq h }\right] t1\left[h\right] VOIAC [\frac{MBq}{g }]$$

Since it is well known that:

$${\text{SUV}}=\frac{VOIAC\left[\frac{MBq}{g }\right] BW[g] {2}^{\frac{t1}{{T}_{1/2}}}}{ID [MBq]}$$

We rephrased the dose equation in terms of VOI counts. Finally, the max/peak/mean dose in the VOI using max/peak/mean SUVs was:

$$D\left[Gy\right]=0.25 \left[Gy \frac{g}{MBq h }\right] t1\left[h\right] \frac{SUV ID[MBq]}{BW \left[g\right] {2}^{\frac{t1}{{T}_{1/2}}}}$$

where *D* is TAD, VOIAC stands for VOI activity concentration, t1 is SPECT/CT acquisition time from injection [h], BW is body weight [g], ID is the injected dose [MBq], and T_1/2_ is the half-life of ^177^Lu [h]. Notably, the dose was based on decay-uncorrected VOI counts, whereas SUV was based on decay-corrected VOI counts. In addition, we also employed the assumptions of the OLINDA unit-density sphere model and no cross dose between organs as conducted by Hanscheid et al. [14].

The cumulative tumour-absorbed Dose_max_, Dose_peak_, and Dose_41_ were defined as the sum of the tumour-absorbed Dose_max_, Dose_peak_, and Dose_41_ from all PRRT cycles, respectively. The cut-off values of the cumulative tumour-absorbed Dose_max_, Dose_peak_, and Dose_41_ to achieve disease control were checked for all target lesions.

### Inter-cyclic changes in TAD

The ratio of the TAD between PRRT cycles (R_N, M_) was calculated as follows:

*R*_*N,M*_ (%) = 100 (TAD from PRRT cycle *M*/TAD from PRRT cycle *N*) (*N* = 1–3, *M* = 2–4, *M* > *N*). The TADs estimated from target lesions with all four cycles of PRRT and SPECT/CT were used to calculate the inter-cyclic changes. Inter-cyclic changes of TAD were used to extrapolate missing SUV data.

### Statistical analysis

Commercially available software, SPSS for Windows (version 21.0; IBM, Chicago, USA), was used to conduct statistical analyses. The correlations between diameter change of the target lesion (%) and the cycle 1 SUV, cycle 1 TAD, and cumulative TAD were evaluated using the Pearson correlation coefficient (*r*). Fisher z transformation was performed to compare correlation coefficients. The target lesion response was divided into two categories, namely disease control and disease progression, and a binary logistic regression method was used to explain the relationship between the TAD and target lesion response. A *p*-value < 0.05 was considered statistically significant.

## Results

### Patients and PRRT

Finally, 20 patients [6 men and 14 women; mean ± standard deviation (SD) age: 57.5 ± 9.6 years, range: 34–75 years] were included in this retrospective study. The primary tumour sites were the pancreas, rectum, duodenum, stomach, kidney, and unknown in 10, 6, 1, 1, 1, and 1 patients, respectively. Hepatic metastases were detected in all patients. Among the extrahepatic metastases, lymph node metastases, bone metastases, peritoneal seeding, and other metastases were detected in 15, 11, 3, and 5 patients, respectively. The Ki-67 index of histopathologically confirmed tumours was ≤ 2%, 3–20%, and > 20% in 1, 15, and 4 patients, respectively. The Krenning scores of tumours with the most intense uptake on pre-therapeutic [^68^Ga]Ga-DOTA-TOC PET/CT were three in six patients and four in 14 patients. Among the 55 target lesions, 7, 37, 10, and 1 target lesions were in the pancreas, liver, lymph node, and peritoneal seeding, respectively. All patients received at least two cycles of PRRT; however, most received four. The interval between PRRT cycles was 71 ± 19 days (range: 49–151 days). The patient characteristics are summarised in Table 1. Most patients (85%) did not receive other treatments, except for short-acting somatostatin analogue approximately 1 month before and after PRRT. However, three patients (15%) received everolimus and PRRT concomitantly (Table 2). SPECT/CT imaging was not performed in six out of 75 PRRT cycles for different patients. Thirteen SUV data from different target lesions could not be measured as these data were extrapolated using inter-cyclic changes between PRRT cycles. The detailed number of target lesions and SUV data are listed in Additional file 1: Table S1, and the volumes of the target lesions are listed in Additional file 1: Table S2.

**Table 1:** Patient demographics and baseline clinical characteristics (*n* = 20)

Variables	Values
Age at diagnosis (years)	57.5 ± 9.6 [34–75]
Sex (male/female)	6:14
Primary tumour site, *n* (%)	
Pancreas	10 (50%)
Rectum	6 (30%)
Duodenum	1 (5%)
Stomach	1 (5%)
Kidney	1 (5%)
Unknown	1 (5%)
Hepatic metastasis, *n* (%)	20 (100%)
Extrahepatic metastasis, *n* (%)	18 (90%)
Lymph node	15 (75%)
Bone	11 (55%)
Peritoneal seeding	3 (15%)
Other	5 (25%)
Grade, n (%)	
1	1 (5%)
2	15 (75%)
3	4 (20%)
Ki-67, n (%)	
< 3%	1 (0.7%)
3–20%	15 (9.5% ± 4.8%)
> 20%	4 (25.3% ± 3.3%)
Krenning score, n (%)	
3	6 (30%)
4	14 (70%)
Site of target lesions, n	55
Pancreas	7
Liver	37
Lymph node	10
Peritoneal seeding	1
Number of PRRT cycles, *n* (%)	
1	0 (0%)
2	2 (10%)
3	1 (5%)
4	17 (85%)
Interval between PRRT cycles (days)	71 ± 19 [49–151]

SD: standard deviation, PRRT: peptide receptor radionuclide therapy

**Table 2:** Patient treatment other than PRRT (*n* = 20)

Treatment	Number of patients, n (%)
Previous treatment	
Surgery	11 (55%)
Liver-directed treatment	5 (25%)
RFA	2 (10%)
TACE	3 (15%)
Somatostatin analogue	13 (65%)
Cytotoxic chemotherapy	14 (70%)
Everolimus	16 (80%)
Concomitant treatment	
Everolimus	3 (15%)

RFA: radiofrequency ablation, TACE: transarterial chemoembolization

### Cyclic changes in the TAD

Cyclic changes in the TAD were calculated using 34 target lesions from 12 patients who received all four cycles of PRRT after correction for administered [^177^Lu]Lu-DOTA-TATE activity. The TAD tends to decrease gradually after each PRRT cycle. The cyclic changes in the TAD are summarised in Additional file 1: Table S3.

### TAD and diameter change of the target lesion relationship

The SLD of target lesions on pre-therapeutic CT was 104.2 ± 54.9 mm (range between the 25th and 75th percentiles: 69.5–128.4 mm). The change in the SLD of target lesions was 17.7 ± 28.4 mm (range between the 25th and 75th percentiles: 7.5–32.1 mm) and 12.6% ± 32.2% (range between the 25th and the 75th percentiles: 5.6%–31.1%). The median cumulative TAD_max_, TAD_peak_, and TAD_41_ of the patients were 127.5 Gy (range between the 25th and 75th percentiles: 70.9–190.6 Gy), 113.9 Gy (range between the 25th and 75th percentiles: 64.4–171.9 Gy), 77.9 Gy (range between the 25th and 75th percentiles: 43.4–117.7 Gy), respectively.

The LD of the target lesion on pre-therapeutic CT was 37.9 ± 20.6 mm (range between the 25th and 75th percentiles: 24.0–44.7 mm). The diameter change of the target lesion was 6.4 ± 12.1 mm (range between the 25th and 75th percentiles: 2.8–13.3 mm) and 15.2% ± 29.4% (range between the 25th and 75th percentiles: 7.9%–31.2%). The cumulative TAD_max_, TAD_peak_, and TAD_41_ of the total lesions were 122.4 Gy (range between the 25th and 75th percentiles: 63.0–181.7 Gy), 112.3 Gy (range between the 25th and 75th percentiles: 56.1–166.2 Gy), and 70.6 Gy (range between the 25th and 75th percentiles: 37.3–111.3 Gy), respectively. Details regarding the cycle 1 and cumulative TADs of the target lesions based on patient-based and lesion-based analyses are summarised in Table 3.

**Table 3:** Cycle 1 and cumulative TADs of the target lesions

	Per-patient	Total lesion	Pancreas	Liver	Lymph node
Cycle 1 TAD_max_ (Gy)	42.3 [5.5–120.3] IQR: 21.9–76.4	38.9 [3.7–179.5] IQR: 19.8–69.8	32.7 [8.6–84.8] IQR: 23.1–59.2	46.5 [3.7–179.5] IQR: 32.5–79.1	19.7 [12.2–38.9] IQR: 15.0–26.6
Cycle 1 TAD_peak_ (Gy)	38.2 [5.0–108.6] IQR: 19.9–69.5	33.7 [3.3–167.1] IQR: 17.9–64.3	30.2 [7.5–78.8] IQR: 21.6–55.4	41.6 [3.3–167.1] IQR: 27.3–73.5	17.4 [10.9–33.7] IQR: 13.6–23.8
Cycle 1 TAD_41_ (Gy)	26.1 [3.6–74.8] IQR: 13.2–46.8	23.8 [2.3–110.5] IQR: 12.5–42.7	18.6 [5.7–47.5] IQR: 14.8–35.3	28.4 [2.3–110.5] IQR: 19.6–44.5	12.5 [7.6–23.8] IQR: 9.4–16.4
Cumulative TAD_max_ (Gy)	127.5 [36.6–271.9] IQR: 70.9–190.6	122.4 [17.6–329.7] IQR: 63.0–181.7	110.3 [26.0–234.9] IQR: 69.1–179.9	149.6 [17.6–329.7] IQR: 91.2–214.8	55.6 [28.8–90.0] IQR: 44.7–69.5
Cumulative TAD_peak_ (Gy)	113.9 [33.8–253.7] IQR: 64.4–171.9	112.3 [14.5–298.2] IQR: 56.1–166.2	101.3 [23.3–217.8] IQR: 64.1–166.2	132.4 [14.5–298.2] IQR: 86.4–187.9	48.3 [26.9–79.3] IQR: 40.0–61.2
Cumulative TAD_41_ (Gy)	77.9 [23.8–170.2] IQR: 43.4–117.7	70.6 [10.9–208.7] IQR: 37.3–111.3	62.9 [16.2–142.0] IQR: 43.2–107.3	91.0 [10.9–208.7] IQR: 57.9–130.2	34.4 [18.2–55.5] IQR: 28.3–42.0

Median [Range]

TAD: tumour-absorbed dose, IQR: interquartile range,

Per-patient: weighted average of the TADs of the target lesions in every patient, Cumulative TAD: sum of the TADs from all PRRT cycles

Based on the RECIST 1.1 criteria, 7, 11, and 2 patients were classified as partial response, stable disease, and progressive disease, respectively. Based on the RECIST-L criteria, 15, 33, and 7 target lesions were classified as partial response, stable disease, and progressive disease, respectively (Table 4).

**Table 4:** Patient response summary and target lesions

	Response		
	Partial response, *n*	Stable disease, *n*	Progressive disease, *n*
Overall patients (*n* = 20)	7	11	2
Total lesion (*n* = 55)	15	33	7
Pancreas (*n* = 7)	0	5	2
Liver (*n* = 37)	12	22	3
Lymph node (*n* = 10)	3	5	2
Peritoneal seeding (*n* = 1)	0	1	0

Neither the cycle 1 SUV nor the cycle 1 TAD was significantly correlated with changes in the SLD or LD of the target lesion (%). The cumulative TAD_max_ (*r* = 0.428, *p* = 0.060), TAD_peak_ (*r* = 0.419, *p* = 0.066), and TAD_41_ (*r* = 0.424, *p* = 0.063) were moderately correlated with changes in the SLDs of target lesions, however, these correlations were not statistically significant. The cumulative TAD_max_ (*r* = 0.301, *p* = 0.025), TAD_peak_ (*r* = 0.299, *p* = 0.026), and TAD_41_ (*r* = 0.299, *p* = 0.027) were weakly correlated with changes in the LD of target lesions with significance (Table 5). On comparing the r values using Fisher’s z transformation, none of the results were statistically significant. A subgroup analysis without the outlier (a patient with a diameter change of target lesion − 103%), demonstrating similar results, is presented in Additional file 1: Table S4.

**Table 5:** Correlation analyses of cycle 1 SUVs, cycle 1 TADs, and cumulative TADs with diameter change (%)

	*r *(patient-based)	*p* (patient-based)	Durbin–Watson (patient-based)	*r *(lesion-based)	*p* (lesion-based)	Durbin–Watson (lesion-based)
Cycle 1 SUV_max_	0.313	0.178	1.483	0.198	0.147	1.866
Cycle 1 SUV_peak_	0.316	0.175	1.486	0.206	0.131	1.869
Cycle 1 SUV_41_	0.313	0.178	1.476	0.201	0.141	1.867
Cycle 1 TAD_max_ (Gy)	0.327	0.160	1.527	0.209	0.126	1.86
Cycle 1 TAD_peak_ (Gy)	0.332	0.153	1.532	0.217	0.112	1.863
Cycle 1 TAD_41_ (Gy)	0.326	0.161	1.519	0.21	0.123	1.861
Cumulative TAD_max_ (Gy)	0.428	0.060	1.668	0.301	0.025*	1.866
Cumulative TAD_peak_ (Gy)	0.419	0.066	1.658	0.299	0.026*	1.873
Cumulative TAD_41_ (Gy)	0.424	0.063	1.653	0.299	0.027*	1.869

SUV: standardised uptake value, TAD: tumour-absorbed dose

Cumulative TAD: sum of the tumour-absorbed doses from all PRRT cycles

A subgroup analysis without the outlier is presented in Additional file 1: Table S4

*p < 0.05

**Fig. 2:**
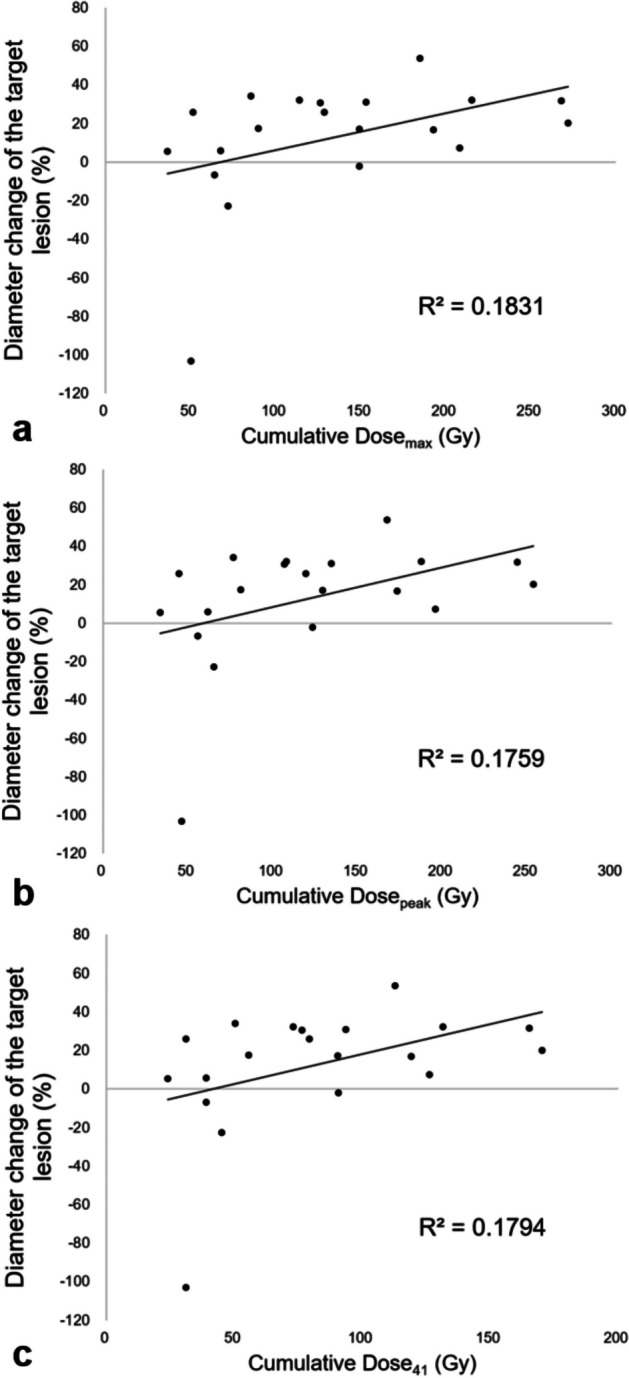
Patient-based scatter plots of the cumulative tumour-absorbed dose (TAD) against the diameter change of the target lesion (%). **A** Cumulative TAD_max_, **B** Cumulative TAD_peak_, and **C** Cumulative TAD_41_

**Fig. 3:**
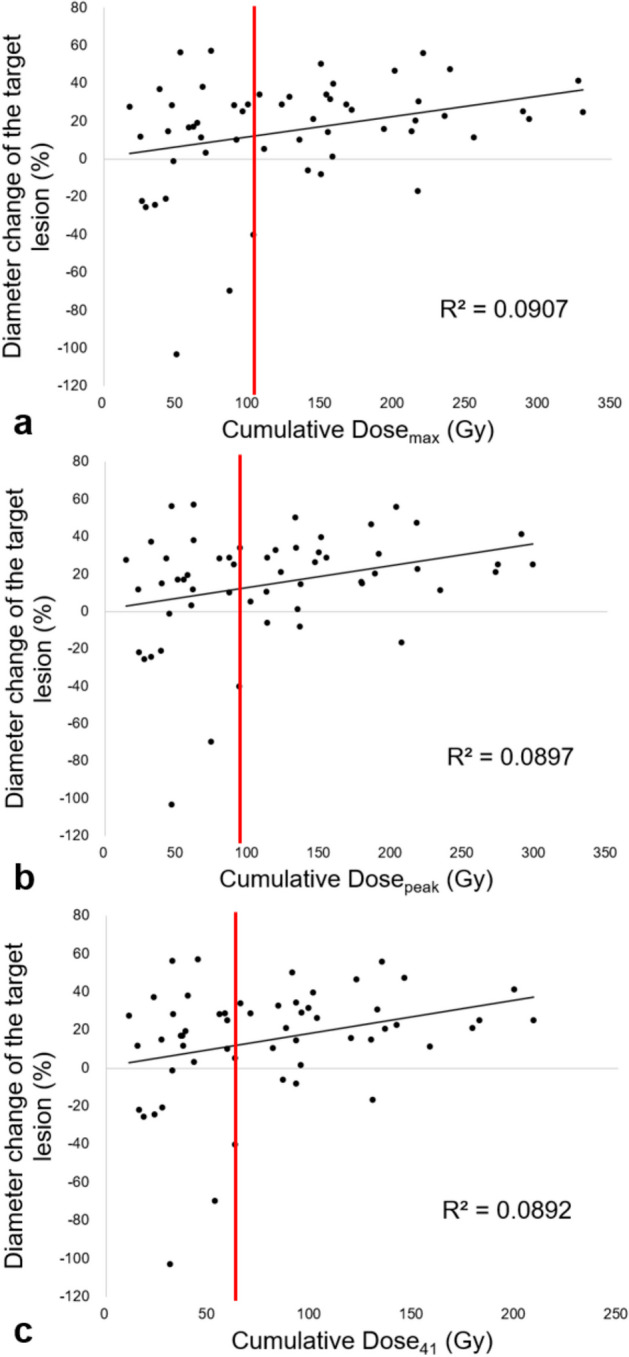
Lesion-based scatter plots of the cumulative tumour-absorbed dose (TAD) against the diameter change of the target lesion (%). **A** Cumulative TAD_max_: Diameter change (%) of the target lesion was > –20% (disease control state according to RECIST 1.1 criteria) when the cumulative dose was ≥ 107.4 Gy (vertical red line). **B** Cumulative TAD_peak_: Diameter change (%) of the target lesion was > –20% (disease control state according to RECIST criteria) when the cumulative dose was ≥ 93.7 Gy (vertical red line). **C** Cumulative TAD_41_: Diameter change (%) of the target lesion was > –20% (disease control state according to RECIST criteria) when the cumulative dose was ≥ 65.4 Gy (vertical red line)

Patient- and lesion-based scatter plots of the cumulative TAD against the diameter change of the target lesion are shown in Figs. 2 and 3, respectively. The change in the LD of the target lesion exceeded –20% (‘disease control’ state according to the RECIST-L criteria) when the cumulative Dose_max_, Dose_peak_, and Dose_41_ were ≥ 107.4, 93.7, and 65.4 Gy, respectively (Fig. 3).

**Fig. 4:**
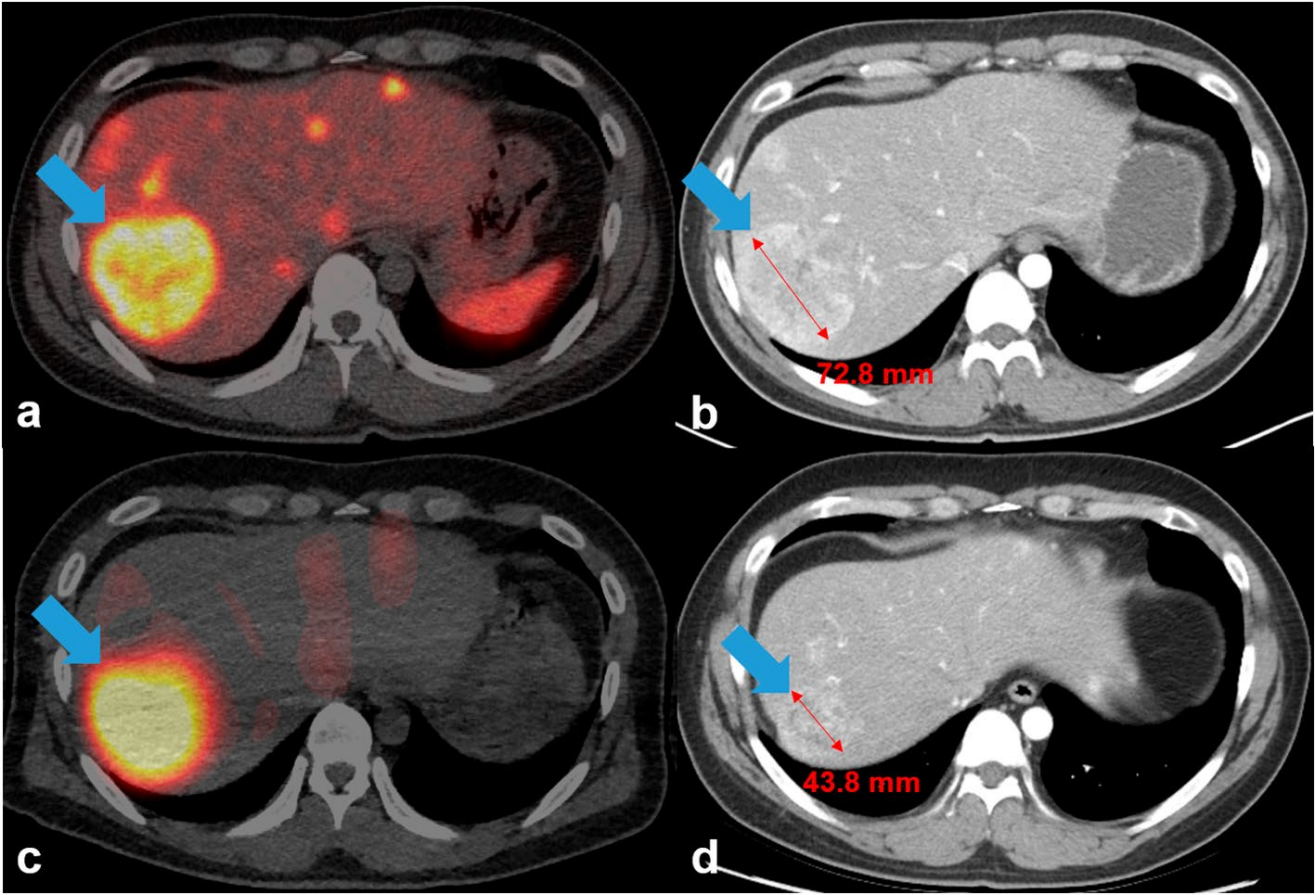
Representative images of the patient’s target lesion (liver) following [^177^Lu]Lu-DOTA-TATE therapy, showing a partial response (arrows). **A** Pre-therapeutic [^68^Ga]Ga-DOTA-TOC PET/CT exhibited a target lesion uptake in the liver that was more intense than normal liver uptake (Krenning score 3). **B** The lesion’s largest diameter measured 72.8 mm on pre-therapeutic CECT. After four cycles of [^177^Lu]Lu-DOTA-TATE therapy, **C** [^177^Lu]Lu-DOTA-TATE SPECT/CT, captured after the fourth PRRT cycle (SPECT/CT images after PRRT cycles 1–3 are not shown), revealed target lesion uptake in the liver that was more intense than normal liver uptake (Krenning score 3). The cumulative tumour-absorbed Dose_max_, Dose_peak_, and Dose_41_ of the target lesions were 157.8, 150.7, and 101.2 Gy, respectively. Following PRRT, the target lesion’s longest diameter decreased by > 30%, measuring 43.8 mm on (**D**) post-therapeutic CECT

Binary logistic regression analysis was performed to determine the relationship between the cycle 1 SUV, cycle 1 TAD, and cumulative TAD and disease control according to the RECIST 1.1 or RECIST-L criteria. The only statistically significant odds ratio observed was between the cumulative TAD and disease control, as per the RECIST-L criteria. Based on the RECIST-L criteria, the probability of disease control increased by 3.1% [95% confidence interval (CI): 0.4%, 5.9%], 3.4% (95% CI: 0.4%, 6.6%), and 5.1% (95% CI: 0.6%, 9.8%) as the cumulative TAD_max_, TAD_peak_, and TAD_41_ increased by 1 Gy, respectively (Table 6). A representative case of partial response is presented in Fig. 4.

**Table 6:** Binary logistic regression analyses between cumulative TADs and target lesion response based on RECIST-L criteria

Dependent variable	Independent variable	Odds ratio [confidence interval]	*p*
Disease control (RECIST-L)	Cumulative TAD_max_	1.031 [1.004, 1.059]	0.024*
	Cumulative TAD_peak_	1.034 [1.004, 1.066]	0.025*
	Cumulative TAD_41_	1.051 [1.006, 1.098]	0.026*

TAD: tumour-absorbed dose

Cumulative TAD: sum of the tumour-absorbed doses from all PRRT cycles

^*^p < 0.05

## Discussion

Our research demonstrated a significant correlation between the cumulative TAD and percentage changes in LD of the target lesion [30]. The correlation between the cumulative TAD and percentage changes in the SLD of the patient was moderate but not statistically significant. There was no significant correlation between the LD of the target lesion and the cycle 1 SUV or TAD. The analysis excluding outliers similarly indicated a significant correlation only between the cumulative TAD and the percentage changes in the LD of the target lesion. Binary logistic regression analysis showed that an increase in the cumulative TAD would lead to a greater chance of disease control based on the RECIST-L criteria.

In our study, every target lesion achieved disease control when the cumulative TAD_max_, cumulative TAD_peak_, and cumulative TAD_41_ were not < 107.4, 93.7, and 65.4 Gy, respectively. These cumulative TADs of target lesions could serve as potential threshold values to anticipate favourable treatment responses during PRRT. However, it should be noted that only two out of 20 patients in our study cohort did not achieve disease control.

On analysing the correlation between cycle 1 TADs and cumulative TADs, *R*^2^ ranged from 0.77 to 0.79 (strong correlation; cycle 1 TAD_max_ vs. cumulative TAD_max_: *R*^2^ = 0.78, cycle 1 TAD_peak_ vs. cumulative TAD_peak_: *R*^2^ = 0.79, cycle 1 TAD_41_ vs. cumulative TAD_41_: *R*^2^ = 0.78), implying that cycle 1 TADs could not be used to fully estimate cumulative TADs. In contrast, *R*^2^ between cumulative TADs ranged from 0.99 to 1.00 (almost perfect correlation; cumulative TAD_max_ vs. cumulative TAD_peak_: *R*^2^ = 1.00, cumulative TAD_max_ vs. cumulative TAD_41_: *R*^2^ = 0.99, cumulative TAD_peak_ vs. cumulative TAD_41_: *R*^2^ = 1.00), indicating no significant change between cumulative TAD parameters. Moreover, consistent administration of 7.4 GBq in each PRRT cycle resulted in an observed decline in the median TAD over successive cycles. This observation suggests that even when SUVs with comparable intensities are evident in [^68^Ga]Ga-DOTA-TOC PET/CT and [^177^Lu]Lu-DOTA-TATE SPECT/CT across different PRRT cycles, the estimated TAD is potentially reduced in subsequent cycles. Based on these findings, a higher dose of radiotracer, within the patient’s tolerance range, during the initial cycles may enhance therapeutic efficacy.

On comparing our study to previous studies on the dosimetry and/or dose–response relationship of [^177^Lu]Lu-DOTA-TATE treatment, not only our study but also most other studies, except for that by Ilan et al. [11], were retrospective in nature [12, 13, 31–33]. Notably, Ilan et al. [11], Jahn et al. [12], Jahn et al. [31], and Roth et al. [32] specifically included tumours of sizes larger than a certain diameter or volume in their analyses to mitigate the partial volume effect, whereas our study and that by Alipour et al. [33] did not. Ilan et al.[11], Jahn et al. [12], and Jahn et al. [31] used evaluation criteria based on the ‘best response’. Conversely, Del Prete et al. [13] and Alipour et al. [33] did not use the ‘best response’ as their criterion. Jahn et al. [12] indicated a slightly weaker lesion-based correlation of *R*^2^ = 0.16 than that in our study, terming it ‘borderline’. A study by Ilan et al. [11] demonstrated a strong lesion-based correlation for lesions with diameters > 2.2 cm (*R*^2^ = 0.64) and > 4 cm (*R*^2^ = 0.91). Jahn et al. [31] further categorised their findings by lesion type, revealing that pancreatic neuroendocrine neoplasms had a correlation of *R*^2^ = 0.37, while small intestine neuroendocrine neoplasms had *R*^2^ = 0.29. Del Prete et al. [13] and Alipour et al. [33] found no significant correlation between dose and volume. The results of the relationship between the TAD and tumour response are potentially affected by various factors, including neuroendocrine neoplasm type and evaluation method, that is, whether it is based on diameter or volume change or whether it uses the ‘best response’ criterion. Comparisons with previous studies on the dose–response relationship can be found in Additional file 1: Table S5.

Regarding the absorbed dose, the median total lesion cycle 1 TAD_41_ determined in our study was 23.8 Gy (range: 2.3–110.5 Gy) with interquartile range of 12.5–42.7 Gy. In comparison, Ilan et al. [11] reported the most frequent cycle 1 TAD values around 20 Gy, with a median of 50 Gy (range: 10–170 Gy). Jahn et al. [12] reported a median cycle 1 TAD of 33.51 Gy (range: 11.24–108.5 Gy), with interquartile range of 23.2–51.1 Gy. Roth et al. [32] reported median cycle 1 TADs of 33 Gy for grade 1 tumours and 27 Gy for grade 2 tumours. Lastly, Alipour et al. [33] reported a median cycle 1 TAD of 29 Gy (range: 5–135 Gy) for measurable lesions, using single time point dosimetric measurement (at 24-h post-therapy). There are no significant differences between the cycle 1 TAD values of previous studies and those of our study.

We chose not to discard missing SUV data and instead extrapolated these using inter-cycle changes between PRRT cycles; this approach is more applicable to real-world situations where performing SPECT/CT may not always be feasible. We encountered cases where SPECT/CT was not performed due to various circumstances, such as a patient’s poor condition or personal schedule, leading to 13 cases of unmeasurable SUV data. To address this issue, we calculated the inter-cycle changes in TAD, which we then used to extrapolate the missing SUV data, although the ratio between TADs in the initial and final PRRT cycles may vary by tumour type [31, 34]. We observed a steady decline in the median TAD following each PRRT cycle, consistent with the findings of previous studies [31–33]. While Jahn et al. did not specify any declining values [31], our study found a median TAD_41_ decrease of 14.9–19.8% per cycle. This rate is in line with the reported decline of 14% per cycle for grade 2 tumours by Roth De et al. [32] and 18–25.8% per cycle for grade 1–3 tumours by Alipor et al. [33]. Notably, 61% of the tumours in the study by Alipor et al. [33] were grade 2, which is comparable to our study, where most patients (75%) were classified as grade 2.

We evaluated up to five target lesions per patient according to the RECIST 1.1 and practical PERCIST 1.0 guidelines and conducted both patient-based and lesion-based analyses. Some studies simplify their design by selecting a single target lesion with the highest uptake per patient when using [^68^Ga]Ga-DOTA-TOC PET/CT to evaluate the treatment response of [^177^Lu]Lu-DOTA-TATE [35]. However, considering the inherent heterogeneity of NETs, the evaluation of multiple lesions per patient would provide a more comprehensive reflection of tumour characteristics [36].

The TAD was generally thought to be estimated from the SUV_mean_ (in our study, the cumulative TAD_41_); however, our study yielded similar results for the cumulative TAD_max_ and cumulative TAD_peak_. As the cumulative TAD_max_ and cumulative TAD_peak_ offer the advantage of simple and reproducible measurements without the need for specific software, such as MIRADA, for assessment, these parameters could be widely applied in future studies.

A SPECT/CT schedule of 4–5 days after PRRT is preferable for patient convenience compared with that of 7 days. However, some reports have suggested that the absorbed dose conversion using 7-day data is more accurate than that using 4–5-day data [14, 15, 37]. Further studies would be needed to compare the TAD using 4–5- and 7-day post-PRRT data.

The median time interval of 30 days (range: 2–71 days) between the final PRRT cycle and post-therapeutic CT in our study was relatively short compared with prior reports of response assessment. This short interval could increase the possibility of pseudo-progression and the underestimation of tumour diameter changes. However, some patients’ early follow-up CT were necessitated by their clinical circumstances. For example, a patient with a − 22.8% change in diameter (who demonstrated disease progression) underwent follow-up CT only 2 days after the last PRRT cycle due to our clinical suspicion of disease progression.

Several limitations should be considered when applying the results of this study to real-world scenarios. First, this study followed a retrospective design and was conducted using a small cohort. Therefore, the results should not be over-emphasised. In addition, 11 out of 20 patients were diagnosed with bone metastases; however, bone lesions were not considered target lesions in our study. However, it is widely recognised that evaluating treatment response by measuring changes in target lesion size on CT scans has limitations [38]. Furthermore, as we did not use partial volume effect correction methods [39], the mean TAD could have been underestimated. Finally, we were unable to analyse the correlation between the disease control status of target lesions and clinical outcomes, such as mortality. Therefore, further research is required to explore the clinical significance and implications of our findings.

## Conclusions

The cumulative TAD estimated from [^177^Lu]Lu-DOTA-TATE SPECT/CT conducted 4–5 days after PRRT demonstrated significant correlations with changes in the LD of the target lesion in per-lesion analyses. These correlations with the cumulative TAD were found to be stronger than that with the cycle 1 SUV or TAD. Furthermore, a higher cumulative TAD was associated with a higher likelihood of disease control in the target lesion. Notably, cumulative TAD_max_ showed a correlation that was at least as robust as cumulative TAD_peak_ and cumulative TAD_41_, suggesting its potential use as a convenient and valuable parameter for predicting tumour response after PRRT. Nonetheless, considering the constraints of the limited sample in this study, a cautious approach to these results is advised.

### Supplementary Information

**Additional file 1. Table S1.** Details of the number of target lesions and SUV data. **Table S2.** Volume of the target lesions. **Table S3.** Ratios of tumour-absorbed doses (TADs) between PRRT cycles. **Table S4.** Correlation analyses of cycle 1 SUVs, cycle 1 TADs, and cumulative TADs with diameter change (%) without the outlier. **Table S5.** Comparisons with previous studies on the relationship between tumour-absorbed dose (TAD) and response.

## Data Availability

The datasets generated during and/or analysed during the current study are available from the corresponding author on reasonable request.

## Abbreviations

DOTA-TATE: DOTA-0-Tyr3-Octreotate
PRRT: Peptide receptor radionuclide therapy
SSTR: Somatostatin receptor
NET: Neuroendocrine tumour
SPECT: Single-photon emission computed tomography
CT: Computed tomography
DOTA-TOC: DOTA-D-Phe1-Tyr3-Octreotide
PET: Positron emission tomography
CECT: Contrast-enhanced CT
KPS: Karnofsky performance status
eGFR: Estimated glomerular filtration rate
Hb: Haemoglobin
WBC: White blood cell
PLT: Platelet
IV: Intravenous
SpO_2_: Saturation pulse oxygen
OSCGM: Ordered subset conjugate gradient minimiser
SLD: Sum of the longest diameters
LD: Longest diameter
SUV: Standardised uptake value
VOI: Volume-of-interest
TAD: Tumour-absorbed dose
BW: Body weight
ID: Injected dose
CI: Confidence interval
