# Supplementary material for: Prediction of [177Lu]Lu-DOTA-TATE therapy response using the absorbed dose estimated from [177Lu]Lu-DOTA-TATE SPECT/CT in patients with metastatic neuroendocrine tumour
Source: EJNMMI Phys. 2024 Feb 5;11:14. doi: 10.1186/s40658-024-00620-8 (PMC10844176; doi:10.1186/s40658-024-00620-8)
Supplement: Supplementary file 1 — Additional file 1. Table S1. Details of the number of target lesions and SUV data. Table S2. Volume of the target lesions. Table S3. Ratios of tumour-absorbed doses (TADs) between PRRT cycles. Table S4. Correlation analyses of cycle 1 SUVs, cycle 1 TADs, and cumulative TADs with diameter change (%) without the outlier. Table S5. Comparisons with previous studies on the relationship between tumour-absorbed dose (TAD) and response. [file 40658_2024_620_MOESM1_ESM.docx]

**SUPPLEMENTARY TABLES**

**Supplementary Table 1.** Details of the number of target lesions and SUV data

| Number of PRRT cycles | Number of target lesions | Total number of SUV datasets | Number of SUV datasets without SPECT/CT |
| --- | --- | --- | --- |
| 1 | 0 | 0 | 0 |
| 2 | 8 | 16 | 0 |
| 3 | 1 | 3 | 1 |
| 4 | 46 | 184 | 12 |
| Total | 55 | 203 | 13 |

SUV: standardised uptake value, PRRT: peptide receptor radionuclide therapy, SPECT/CT: single-photon emission computed tomography/computed tomography

**Supplementary Table 2.** Volume of the target lesions

|  | Cycle 1 | Cycle 2 | Cycle 3 | Cycle 4 |
| --- | --- | --- | --- | --- |
| Total lesion (cm^3^) | 14.6 [3.6–127.8] | 13.9 [4.3–303.1] | 12.0 [3.6–176.2] | 12.0 [3.0–190.8] |
| Pancreas (cm^3^) | 30.1 [7.1–46.1] | 23.5 [13.9–101.7] | 22.5 [12.5–86.1] | 21.0 [14.5–65.3] |
| Liver (cm^3^) | 15.8 [3.6–127.8] | 15.4 [4.3–303.1] | 11.3 [3.6–176.2] | 12.0 [3.0–190.8] |
| Lymph node (cm^3^) | 10.5 [6.9–42.6] | 10.0 [5.6–29.6] | 9.2 [5.9–24.6] | 8.7 [5.9–24.8] |
| Median [range] | | | | |

**Supplementary Table 3.** Ratios of tumour-absorbed doses (TADs) between PRRT cycles

| Cycles of PRRT | TAD_max_ | TAD_peak_ | TAD_41_ | Mean |  |
| --- | --- | --- | --- | --- | --- |
| *R_1,2_* | 90.8 ± 41.0 [81.3]% | 90.0 ± 41.6 [80.5]% | 90.2 ± 38.7 [80.2]% | 90.4% |  |
| *R_2,3_* | 84.1 ± 19.4 [82.8]% | 83.7 ± 19.3 [84.2]% | 84.3 ± 19.1 [85.1]% | 84.1% |  |
| *R_3,4_* | 87.2 ± 19.4 [84.6]% | 87.1 ± 20.3 [84.3]% | 87.8 ± 19.0 [85.0]% | 87.3% |  |
| *R_1,4_* | 63.7 ± 30.9 [55.2]% | 62.9 ± 31.8 [54.3]% | 64.8 ± 32.3 [55.7]% | 63.8% |  |
| PRRT: peptide receptor radionuclide therapy  Mean ± SD [median] | | | | | |

**Supplementary Table 4.** Correlation analyses of cycle 1 SUVs, cycle 1 TADs, and cumulative TADs with diameter change (%) without the outlier

|  | r  (patient-based) | p  (patient-based) | Durbin–Watson  (patient-based) | r  (lesion-based) | p  (lesion-based) | Durbin–Watson  (lesion-based) |
| --- | --- | --- | --- | --- | --- | --- |
| Cycle 1 SUV_max_ | 0.252 | 0.298 | 1.859 | 0.167 | 0.227 | 1.405 |
| Cycle 1 SUV_peak_ | 0.261 | 0.281 | 1.857 | 0.177 | 0.201 | 1.411 |
| Cycle 1 SUV_41_ | 0.255 | 0.293 | 1.848 | 0.171 | 0.217 | 1.408 |
| Cycle 1 TAD_max_ (Gy) | 0.255 | 0.292 | 1.884 | 0.176 | 0.202 | 1.395 |
| Cycle 1 TAD_peak_ (Gy) | 0.267 | 0.269 | 1.880 | 0.186 | 0.178 | 1.400 |
| Cycle 1 TAD_41_ (Gy) | 0.256 | 0.289 | 1.874 | 0.178 | 0.198 | 1.397 |
| Cumulative TAD_max_ (Gy) | 0.379 | 0.109 | 1.828 | 0.274 | 0.045* | 1.556 |
| Cumulative TAD_peak_ (Gy) | 0.377 | 0.112 | 1.837 | 0.274 | 0.045* | 1.554 |
| Cumulative TAD_41_ (Gy) | 0.376 | 0.113 | 1.816 | 0.271 | 0.047* | 1.557 |

SUV: standardised uptake value, TAD: tumour-absorbed dose

Cumulative TAD: sum of the tumour-absorbed doses from all PRRT cycles

*p < 0.05

**Supplementary Table 5.** Comparisons with previous studies on the relationship between tumour-absorbed dose (TAD) and response

| Study | Cohort | Inclusion criteria | Target lesion selection | Number of patients/lesions | Response evaluation criteria | Dose–response findings between cumulative TAD and diameter or volume | Dosimetry |
| --- | --- | --- | --- | --- | --- | --- | --- |
| Ha et al. (Our study) | Retrospective | Metastatic NEN (Krenning score: 3–4) | > 1 cm Up to 5, maximum 2 per organ | 20 patients/55 lesions | RECIST 1.1 3 mo after final PRRT cycle | Diameter change Significant, but weak Lesion-based (R^2^ = 0.09) | Single time-point dosimetric measurements at 96-120 h PRRT (xSPECT/CT) |
| Ilan et al. (2015) | Prospective | Metastasised pancreatic NEN | Diameter > 2.2 cm | 24 patients/24 lesions  (Diameter > 4 cm: 12 lesions) | RECIST 1.1  Best response Before cycle 3, cycle 5 3 mo after final PRRT cycle | Lesion-based diameter change 2-parameter sigmoid fits Diameter > 2.2 cm (R^2^ = 0.64) Diameter > 4 cm (R^2^ = 0.91) | Complete dosimetric evaluation |
| Del Prete et al. (2017) | Retrospective | NEN treated with at least one cycle of PRRT | Diameter > 2 cm | 15 patients/36 lesion | RECIST 1.1 3 mo after cycle 4 PRRT | Lesion-based diameter change No correlation | QSPECT-based dosimetry SPECT/CT: 4, 24, and 72 h |
| Jahn et al. (2020) | Retrospective | Metastases from small-intestinal NEN | Diameter > 2.2 cm For all cycles | 25 patients/25 lesions | RECIST 1.1  Best response Before cycle 3, cycle 5 3, 6, and 12 mo, and yearly after final PRRT cycle | Lesion-based  BR volume: R^2^ = 0.28 BR diameter: R^2^ = 0.16 (borderline significant) | Complete dosimetric evaluation |
| Jahn et al. (2021) | Retrospective | Pancreatic or small-intestinal NEN  (Krenning score: 3–4) | Diameter > 2.2 cm  For all cycles Similar size | 48 patients/48 lesions | RECIST 1.1  Best response Before cycle 3, cycle 5 3, 6, and 12 mo, and yearly after final PRRT cycle | Lesion-based diameter change Significant, but weak Pancreatic NENs: R^2^ = 0.37 Small intestinal NENs: R^2^ = 0.29 | Complete dosimetric evaluation |
| Roth et al. (2022) | Retrospective | GEP or BP NEN | Volume > 8 cm^3^ | 39 patients/109 lesions | Not evaluated | Decreased volume over the cycle Not analysed | Complete dosimetric evaluation |
| Alipour et al. (2023) | Retrospective | Unresectable GEP NEN | Index lesion: most avid lesion Measurable lesion: up to 5, maximum 2 per organ | 90 patients | RECIST 1.1 3 months after final PRRT cycle | Lesion-based Volume (MITVSSR) No correlation | Single time-point dosimetric measurements at 24 h PRRT (Q-SPECT/CT) |

PRRT: peptide receptor radionuclide therapy, SPECT/CT: single-photon emission computed tomography/computed tomography, NEN: neuroendocrine neoplasm, BR: best response, GEP: gastroenteropancreatic, BP: bronchopulmonary, MITVSSR: molecular imaging tumour volume of somatostatin receptor
